# The Multi-Faced Extracellular Vesicles in the Plasma of Chronic Kidney Disease Patients

**DOI:** 10.3389/fcell.2020.00227

**Published:** 2020-04-15

**Authors:** Hara T. Georgatzakou, Efthimia G. Pavlou, Effie G. Papageorgiou, Issidora S. Papassideri, Anastasios G. Kriebardis, Marianna H. Antonelou

**Affiliations:** ^1^Laboratory of Reliability and Quality Control in Laboratory Hematology (HemQcR), Department of Biomedical Sciences, School of Health & Welfare Sciences, University of West Attica, Athens, Greece; ^2^Department of Biology, School of Science, National and Kapodistrian University of Athens (NKUA), Athens, Greece

**Keywords:** CKD, ESRD, extracellular vesicles, dialysis, biomarkers, therapy

## Abstract

Extracellular vesicles (EVs) are membrane-enclosed nanoparticles released by most cells in body fluids and extracellular matrix. They function as signal transducers in intercellular communication, contributing to the maintenance of cell and tissue integrity. EVs biogenesis is deregulated in various pathologies, in structural and functional connection to the pathophysiology of donor cells. Consequently, EVs are considered diagnostic and monitoring factors in many diseases. Despite consensus as to their activity in promoting coagulation and inflammation, there is evidence suggesting protective roles for EVs in stress states. Chronic kidney disease (CKD) patients are at high risk of developing cardiovascular defects. The pathophysiology, comorbidities, and treatment of CKD may individually and in synergy affect extracellular vesiculation in the kidney, endothelium, and blood cells. Oxidative and mechanical stresses, chronic inflammation, and deregulation of calcium and phosphate homeostasis are established stressors of EV release. EVs may affect the clinical severity of CKD by transferring biological response modifiers between renal, vascular, blood, and inflammatory cells. In this Review, we focus on EVs circulating in the plasma of CKD patients. We highlight some recent advances in the understanding of their biogenesis, the effects of dialysis, and pharmacological treatments on them and their potential impact on thrombosis and vascular defects. The strong interest of the scientific community to this exciting field of research may reveal hidden pieces in the pathophysiology of CKD and thus, innovative ways to treat it. Overcoming gaps in EV biology and technical difficulties related to their size and heterogeneity will define the success of the project.

## Extracellular Vesicles: Biovectors and Biomarkers

Extracellular vesiculation is an evolutionary conserved fundamental activity of both normal and diseased cells (Gyorgy et al., 2011). As an integral part of cell growth, aging, or development (Johnstone et al., 1987), as a way to get rid of unneeded cellular material, or as a means of intercellular, interorgan, and interspecies crosstalk (Giricz et al., 2014), extracellular vesiculation is a key homeostatic mechanism contributing to survival, function, remodeling, and repair processes (Bruno et al., 2009). As cells transfer materials inside or outside them by secretory and transport vesicles, absolutely healthy, stressed, or activated cells release nanosized membrane-enclosed extracellular vesicles (EVs) to the external environment. Within blood circulation, EVs have a short half-life (from a couple of minutes to a few hours), before being taken up by neighboring or distant cells.

The highly dynamic and omnipresent EV compartment consists of a heterogeneous group of small exosomes (typically <150 nm), medium sized microvesicles (MVs, or microparticles, typically <1 μm), large sized apoptotic bodies, and several other EV subtypes (Raposo and Stoorvogel, 2013). On a constitutive basis or in response to stimulation, a cell can produce a wide variety of EVs. Their phenotype, biophysical characteristics, and biogenesis may overlap so much between each other and with non-EV components (e.g., plasma protein aggregates) so EVs characterization or classification is often a brainteaser.

Release of membrane vesicles is not a stochastic process but rather a regulated, highly selective cellular ability. It may have negative effects when overactivated, for example, during inflammation. The EVs bear “real-time” molecular signatures of the physiological states of the parental cells and their microenvironment. Based on those compositional and functional correlations, EVs are considered noninvasive predictive, diagnostic, and biomonitoring factors for the development and propagation of several diseases, including cardiovascular events (Sinning et al., 2011) and structural renal injury (Zhou et al., 2006), among others.

Moreover, according to numerous *in vitro* and *in vivo* data, material delivered by plasma EVs to recipient cells may induce immunomodulation, inflammation, oxidative stress, thrombogenesis, and vascular senescence or dysfunction (Gyorgy et al., 2011; Burger et al., 2012; Zecher et al., 2014; Cai et al., 2015; Camus et al., 2015; Hosseinkhani et al., 2018). This is quite reasonable for competent carriers endowed by nature with a remarkable efficiency to preserve their cargo intact and active along their journeys. Secreted cytokines, for example, are more stable inside EVs than free in plasma (Fitzgerald et al., 2018). *Vice versa*, the levels of circulating EVs are increased in diseases associated with inflammation or coagulation (van Beers et al., 2009), while proinflammatory and procoagulant factors promote the extracellular vesiculation by healthy cells *in vitro*.

**FIGURE 1:**
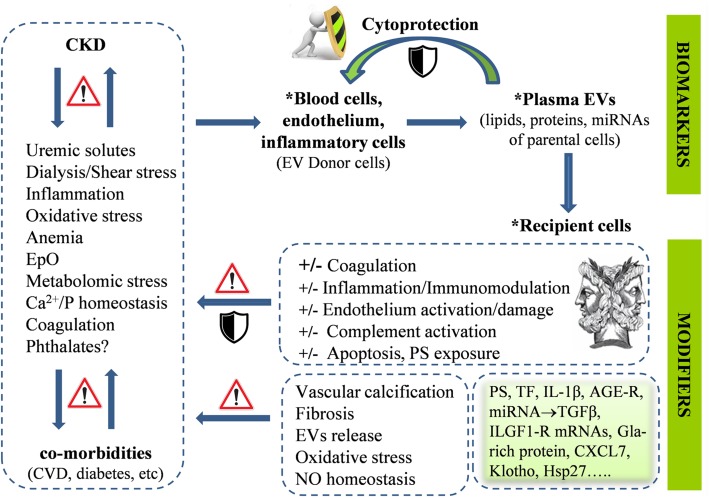
The chronic kidney disease (CKD)-related pathophysiological factors, treatment modalities, and comorbidities affect the cellular stress and activation status of endothelium and blood cells, leading to augmented release of extracellular vesicles (EVs) in the plasma. EVs contain molecular components of parental cells, and thus, are considered biomarkers of disease phenotypes. EV release may represent a cytoprotective mechanism allowing, among other, removal of stress or death signals from donor cells. Interactions of plasma EVs with recipient cells and plasma components may be involved in the progress of the primary disease *per se* and its cardiovascular complications in both directions, either by transmitting and amplifying dangerous, cellular response modifiers or by counteracting them. The “Janus face” is a general feature of EVs functional potential, related to their complex biology, in both donor and recipient cells. Advances in EV research have provided sharper pictures of plasma EVs in CKD, identifying them as key parts of both problem and solution.

Cardiovascular abnormalities represent the leading cause of increased morbidity and mortality in patients with chronic kidney disease (CKD), which may result to end-stage kidney disease (ESKD) (Antonelou et al., 2014). The molecular mechanisms underlining CKD have been associated with a pathophysiological context of chronic inflammation, anemia, accumulation of uremic toxins, increased oxidative and metabolic stresses (Antonelou et al., 2011), deregulation of calcium and phosphate homeostasis, coagulation and vascular abnormalities, and chronic or acute [e.g., by hemodialysis (HD)] endothelial activation (Georgatzakou et al., 2016). Of note, all of these pathologies have also been related to EV biology, either as stress factors triggering EV release or as outcomes of EVs’ effects on sensitive cell targets. Those bidirectional connections reflect the dual role of EVs as both biomarkers of cell dysfunction and vectors of bioactive molecules contributing to it (Figure 1). Not surprisingly, CKD patients have elevated concentration of EVs in body fluids (Amabile et al., 2005).

## EVs in the Plasma of CKD Patients: General Outline of Our Knowledge

Endothelium and blood cells in CKD are characterized by increased rate of extracellular vesiculation. Significant proportion of EVs (in most studies, MVs) that are released following activation of the origin cells (Antonelou et al., 2011) expose phosphatidylserine (PS; Faure et al., 2006; Burton et al., 2013; Yu et al., 2018) and are prothrombotic, often more than the parent cells (Sinauridze et al., 2007), according to both morphological and biochemical evidence (Gao et al., 2015b; Yu et al., 2018). The intriguing finding that the platelet-derived MVs are less procoagulant than in other diseases (Trappenburg et al., 2012) (probably due to functional defects in platelets) does not break up the intimate relation of EVs with thrombosis in CKD, as uremic patients with thrombotic events have more MVs compared to those without events (Ando et al., 2002). Moreover, PS on plasma EVs may nucleate calcium phosphate, contributing to ectopic calcification reactions (Wu et al., 2013). Therefore, pharmacological blocking of the PS binding sites on cells and EVs or lipid lowering drugs (Almquist et al., 2016) have been considered as meaningful measures to prevent thrombosis and vascular calcification risk in CKD. Of note, CKD patients with vascular calcification have both more circulating endothelial MVs and less endothelial progenitor cells compared to patients without calcification. This looming imbalance in endothelial damage and repair processes is probably induced by the MV-mediated expression of osteocalcin (Soriano et al., 2014).

Several cross-talking factors seem to augment extracellular vesiculation in CKD (Figure 1). Uremic toxins constitute the central one. To support, the PS^+^ EV levels *in vivo* are inversely correlated with the glomerular filtration rate (Almquist et al., 2016; Yu et al., 2018), but positively with uric acid and proteinuria levels (Yu et al., 2018). More procoagulant MVs are detected in diabetes mellitus patients with CKD compared to patients without it (Almquist et al., 2016), and in patients with extreme albuminuria than in those with lower or normal levels (Yu et al., 2018). To get a laboratory verification, incubation of normal endothelial cells or red blood cells (RBCs) with (i) patients’ serum, (ii) uremic toxins at concentrations found in patients, or (iii) increased concentration of phosphate induce PS exposure on cells and EVs release (Faure et al., 2006; Abbasian et al., 2015; Gao et al., 2015a, b; Yu et al., 2018).

Another informative (inverse) correlation of endothelial MVs with shear stress is repeatedly detected in ESRD patients. It suggests that variations in shear-stress determinants and blood viscosity, including anemia and hemoconcentration arisen by the dialysis or erythropoietin (EpO) supplementation, may affect the vesiculation rate in the endothelium (Boulanger et al., 2007). Indeed, more endothelial cell-derived MVs have been detected in child and adult dialysis patients with CKD, than in patients not getting dialysis (Dursun et al., 2009; Merino et al., 2010), but those findings may merely reflect the clinical severity of the disease. When comparing patients of similar clinical severity, both inflammation and repetitive mechanical stress, imposed on endothelium and blood cells by hemodialysis, still seem to augment vesiculation (Daniel et al., 2006). The same inducers modify the EV phenotypes and micro-RNA (miRNA) cargos, often in cell-specific (Faure et al., 2006; Trappenburg et al., 2012), or dialysis modal-specific ways (Cavallari et al., 2019). For instance, MV concentration is lower in patients treated by hemodiafiltration versus conventional hemodialysis (Georgatzakou et al., 2018). By examining the short-term effects of a single dialysis session on EV measurements, dialysis still triggers release of MVs but again, with marked differences between high-flux and low-flux dialyzers (de Laval et al., 2019). As to the accumulation of the PS-exposing MVs (Georgatzakou et al., 2018) and smaller EVs (Ruzicka et al., 2019), dialysis generally exerts a beneficial acute effect, mediated in part by EV absorbance to the dialysis membrane (Ruzicka et al., 2019), at least in patients with adequate response to EpO (Georgatzakou et al., 2017). Additionally, hemodialysis seems to be more effective in eliminating small compared to bigger EVs (Ruzicka et al., 2019). There is evidence that EpO affects platelet activation and promotes EV accumulation (Ando et al., 2002). This is quite interesting, because endothelium damage and cardiovascular disease appear more frequently in EpO resistance (Panichi et al., 2011). Despite that, several renal-protective EpO signaling pathways are proceeded through stimulating EV release by both mesenchymal stem cells (Wang et al., 2015) and bone marrow cells (Zhou et al., 2016). Finally, patients on hemodialysis are regularly exposed to considerable amounts of phthalates leaching by plastic tubing (Faouzi et al., 1999). Although the field is aware of the associated risks, the probable contribution of phthalates on comorbidities (Kataria et al., 2017) and extracellular vesiculation [extensively studied in stored blood (Serrano et al., 2016)], as well as their probable dissemination to sensitive targets through EVs, have not been investigated so far in CKD.

## What is the Message of Circulating EVs in CKD?

Like other pathologies, EVs represent phenotypic markers of cellular stress and activation in CKD. More importantly, their number and composition may also play a role in the pathophysiology of cardiovascular complications, and thus in mortality risk (Carmona et al., 2017a).

On one side, a wide panel of “incriminating” bioreactive phenotypes have been detected in plasma EVs of ESRD patients. Apart from PS, those phenotypes include platelet activation markers, tissue factor (TF), IL-1β, miRNAs (Shang et al., 2017), and advanced glycation end products (AGEs) receptor (Stinghen et al., 2016), which are potentially involved in coagulation, inflammation, oxidative stress, and endothelial activation/dysfunction (Stinghen et al., 2016; de Laval et al., 2019). Endothelial dysfunction, arterial stiffness, and atherosclerosis are determinants of cardiovascular risk in patients with or without CKD. Flow cytometry, functional measurements, and Kaplan–Meier survival analysis have showed that the concentration of endothelium-derived MVs in ESRD selectively correlates with arterial dysfunction *in vivo* (Amabile et al., 2005; Dursun et al., 2009), and further predicts cardiovascular mortality (Amabile et al., 2012). MVs released by cells exposed to uremic substances promote functional loss in endothelial progenitor cells (marked by overexpression of NF-κB and p53 proteins) (Carmona et al., 2017b) and TGFβ-mediated proliferation of vascular smooth muscle cells (Ryu et al., 2017), while isolated ESRD MVs can impair NO release and endothelium relaxation (Amabile et al., 2005). Endothelial dysfunction may be further related to activation of the alternative complement pathway and of note, endothelium-derived MVs in CKD do contain factor D and can activate the alternative pathway *in vitro* (Jalal et al., 2018).

Endothelial dysfunction and fibrosis may be promoted in CKD by EV-mediated miRNA transfer mechanisms (Xie et al., 2017). miRNA present in MVs induced by uremic toxins disturb signaling pathways involved in endothelial regeneration through oxidative stress and apoptosis (Carmona et al., 2017b). Indeed, a series of miRNAs that mostly target TGFβ signaling-related mRNAs in recipient cells have been found enriched or depleted in plasma and plasma-derived exosomes and MVs from patients with CKD (Muralidharan et al., 2017). miR-92a, for instance, is involved in atherosclerosis and cardiovascular disease as an effective suppressor of key endothelial-protective mRNAs. High serum levels of miR-92a have been associated with decreased kidney function. Moreover, endothelial MVs of uremic patients are enriched in miR-92a and uremic plasma upregulates its expression in cultured endothelial cells (Shang et al., 2017). Another miRNA, the miR-223 (which is abundant in resting platelets and further upregulated following platelet activation by pro-inflammatory factors), is enriched in platelet MVs of patients with nephritis, or atherosclerosis. It was found that miR-223 can promote apoptosis induced by AGEs in endothelial cells *in vitro* via targeting the insulin-like growth factor 1 receptor (Pan et al., 2014). Of note, a higher expression of miR-223 was found in the endothelial-derived exosomes and MVs of ESRD patients treated by bicarbonate hemodialysis compared to those treated by online hemodiafiltration and healthy subjects in close relation to variation in systemic inflammation markers (Cavallari et al., 2019).

Apart from the EV-delivered miR223, exosomes and MVs of ESRD patients with low levels of the Gla-rich protein (an anti-inflammatory factor and inhibitor of calcification in the cardiovascular system) can induce calcification in target vascular smooth muscle cells, by promoting osteogenic differentiation and inflammation (Viegas et al., 2018). There is evidence that monocyte and endothelial MVs induce podocyte phenotypic modifications *in vitro* [release of proinflammatory cytokines and vascular endothelial growth factor (VEGF)] typically associated with glomerular permeability and proteinuria (Eyre et al., 2011). Moreover, plasma platelet MVs can cause glomerular endothelial injury in animal models of diabetic nephropathy through transferring of chemokine ligand 7 (CXCL7) and activation of the mammalian target of rapamycin complex 1 (mTORC1) pathway in glomerular target cells (Zhang et al., 2018). Finally, recent studies revealed that plasma EVs in ESRD also contain the Klotho protein (de Laval et al., 2019), a transmembrane component of the distal convoluted tubule of the kidney. Klotho expression declines with reducing renal function, and its deficiency has been speculated to contribute to vascular calcification (Hu et al., 2012).

On the other side, EVs release is also an “alarm button” pressed by cells facing life-threatening provocations. In RBCs, for example, extracellular vesiculation increases by aging, oxidative stress, and cold storage-associated lesions (Kriebardis et al., 2008). Proteomic analyses of those EVs have suggested activity toward disposal of abnormal, nonfunctional, or dangerous materials (e.g., complement) that could otherwise signal premature elimination of releasing cells (Willekens et al., 2008; Tzounakas et al., 2016). In a similar way, release of caspase 3-containing EVs by endothelial cells *in vitro* protects them from apoptosis and detachment (Abid Hussein et al., 2007), suggesting that a similar mechanism may contribute to endothelial cell survival in CKD (Figure 1). Consequently, it is reasonable to assume that increased release of PS^+^ RBC-derived EVs in CKD may counterbalance erythrophagocytosis leading to anemia (Georgatzakou et al., 2017). Complete phenotyping of EVs under various clinical and treatment conditions would test the accuracy of this assumption.

In addition, certain EVs may well induce anti-inflammatory responses in recipient cells (Gasser and Schifferli, 2004), although these responses may vary in pathological conditions including hyperglycemia (Jansen et al., 2015). miR-222 (Jansen et al., 2015) and Hsp27 (Shi et al., 2019) laden plasma EVs, for example, were found to reduce tumor necrosis factor-alpha-induced ICAM-1 expression (and monocyte adhesion) and stimulate the release of IL-10 and NF-kappa in endothelial and B cells, respectively, both *in vitro* and *in vivo.* Moreover, plasma MVs that are released by the dysfunctional endothelium of patients with unstable angina are enriched in the miR-19b and exert antithrombotic function by inhibiting TF expression in endothelial cells (Li et al., 2014). Several other miRNAs that are potentially encapsulated in plasma EVs, such as the miR-23b (Zhao et al., 2016), have been shown to alleviate fibrosis and albuminuria in diabetic nephropathy, and thus, their levels are pathologically low in the serum and kidney of the patients. It is worth studying the degree of encapsulation of those cytoprotective miRNAs into the plasma EVs. Recent reports in animal models of kidney disease showed that miRNA expression is lower in plasma-derived exosomes compared to the plasma and that the miRNA levels may widely vary in each disease context (Xie et al., 2017). To sum up, plasma EVs may counteract proinflammatory and procoagulant signals instead of transmitting and amplifying them (Figure 1), and thus, part of them may protect the vascular and kidney functions in CKD.

## How Can We Read the Full Message and Move Forward?

**FIGURE 2:**
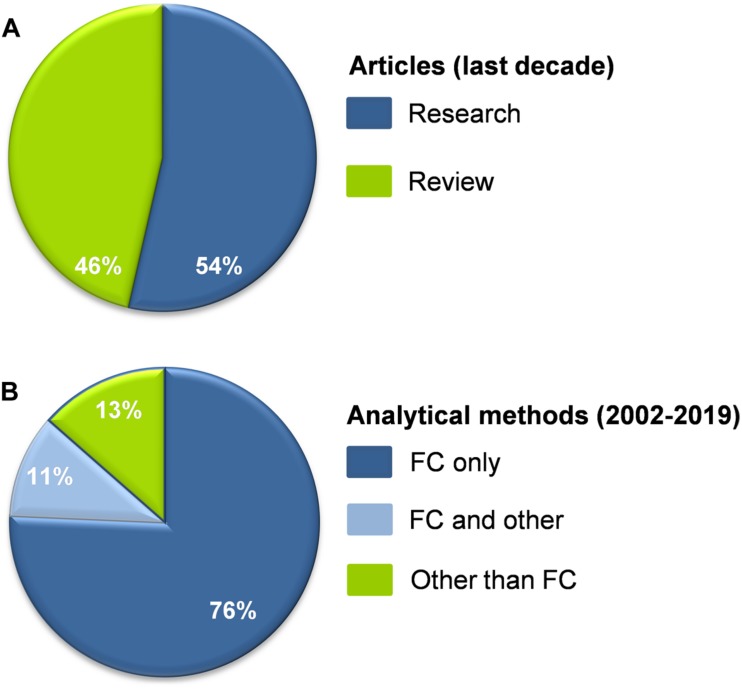
(**(A)** Percentage of research and review articles dealing with plasma EVs in CKD published in the last decade (*N* = 56). **(B)** The vast majority of research data on plasma EVs in CKD derived in the period from 2002 till today (*N* = 37) were based exclusively on flow cytometry (FC). Fewer studies combined FC with other methodologies (immunoblotting, nanoparticle tracking analysis, proteomics, Elisa, electron microscopy) or they did not use FC at all. References are shown in the Supplementary Material.

EVs represent an exciting chapter of contemporary biology and medicine. The disproportionally high ratio of reviews to research articles dealing with plasma EVs in CKD (Figure 2A) reflects a major interest for understanding a subject that is, however, difficult to be studied. Generally, research on EVs is a challenge for the scientific community, mainly due to their size, heterogeneity, artificial generation during the preanalytical stages, and lack of standardized methods of working with them (Doyle and Wang, 2019). Let alone studying them in CKD patients characterized by extreme variabilities in primary lesions, comorbidities, and treatments, which individually and in synergy affect both their release and composition (Carmona et al., 2017a).

Despite that, research on EVs is critical for CKD for many reasons. First of all, we are now aware that EVs are key figures in the frame of this disease. They have to be taken into consideration, for instance, when measuring plasma cytokine levels in patients, because EV-encapsulated cytokines may not be detected by standard cytokine assays (Fitzgerald et al., 2018). Second, EVs have been associated with most of the cardiovascular risk factors (Hosseinkhani et al., 2018) and with poor clinical outcomes in CKD as in many other diseases. Third, regular recording of clinical/laboratory data of CKD patients drives physicians to make individual, intervening decisions on treatments per short intervals of time. Consequently, availability of sensitive tools to support biomonitoring of the disease progression and identification of patients at risk are highly desirable. Finally, similar to their release, EVs uptake is also a specific and regulated process. Since EVs can cross the glomerular basement membrane, the possibility of using them as therapeutic vehicles targeting the kidney cells *per se* (Oosthuyzen et al., 2016) open new prospects for CKD management.

Plasma EVs may provide peripheral biomarkers easily available for clinical diagnosis. The first step toward that is discovery of a reliable marker (or panel of markers) being related to a particular disease state, complication, or pathological condition. The candidate biomarker should then be appropriately validated *in vivo*, in animal disease models at first, and then, in cohorts of patients by clinical studies. Last is bridging of those biomarkers’ capabilities to clinical setting. Isolation of EVs, especially exosomes, is not a clinically friendly procedure; however, development of simple and quick innovative separation methods (like the microfluidic based techniques) provides candidates for potential use in the clinical setting.

Biomarker mining presupposes availability of reliable EV metrical, compositional, physicochemical, and uptake (Zaborowski et al., 2015) data. First of all, analytical methods able to detect and characterize the entire vesicular population, including the smaller (<200 nm) EVs, must be used. The majority of data reported on plasma EVs in CKD have been extracted by flow cytometry, the “gold standard” analytical method for EVs characterization (Figure 2B). However, conventional cytometry cannot detect vesicles < 300 nm, the area that harbors the majority of EVs in human blood (Yuana et al., 2011; Arraud et al., 2014). Apart from that, it is now well established that only few blood EVs expose PS, namely, the most commonly used tag for flow cytometry (Connor et al., 2010; Arraud et al., 2014). Despite insights provided by immobilizing smaller EVs on bead surfaces, and advances in the detection limits of new nanoscale instruments (100–200 nm) (Dragovic et al., 2011; Gomes et al., 2018), the fact is that the biology and functions of smaller plasma EVs are largely unknown in CKD (Kapustin et al., 2015), in opposite to those of urinary exosomes (Erdbrugger and Le, 2016). Of note, however, comparative proteomic analysis of RBC membrane in ESRD patients with good or poor response to EpO administration suggested release of small and/or PS-negative EVs in responders that are undetectable by the conventional flow cytometry (Georgatzakou et al., 2017).

Assessment of vesiculation by the protein concentration of a vesicular pellet is not accurate because pellets contain both disease specific and unspecific protein profiles, and moreover, they are unavoidably contaminated by plasma components (Doyle and Wang, 2019). To get insight into EVs size and concentration, sophisticated physical methods like nanoparticle tracking (Dragovic et al., 2011; Burton et al., 2013) and tunable resistive pulse sensing analyses, in combination with cryo-electron microscopy, are currently used in the EV research field (Hosseinkhani et al., 2018). Compositional analyses are more appropriately performed by immunoblotting, the much informative omics techniques, and by microfluidic devices allowing capture of EVs on a chip’s surface and then lysis and probing of lysate, for identification of both surface and lumen components (He et al., 2014). Thermophoretic enrichment and profiling has been successfully tested for cancer detection and classification as an alternative method to detect surface proteins of plasma EVs, following labeling with fluorescent aptamers (Liu et al., 2019). Under such integrative characterization of plasma EVs, lack of specific protein markers to distinguish between their subtypes becomes less important.

Compositional analyses of EVs are of critical importance to understand the hows and whys of their biogenesis, stimuli, targets, and effects. Global and targeted mass spectrometry-based proteomic analysis is best suitable to suggest biomarker candidates (Zhou et al., 2006), the cellular destinations of EVs, and thus, the most suitable therapeutic targets in a given patient or disease context. However, analysis of one data type by itself is often limited to correlations resulting by reactive processes rather than by causative modifications. Integration of multiomics (Hasin et al., 2017), electron microscopy and physical characterization data (Thery et al., 2018) would rather reveal hidden aspects of EV biology and functions, offering the opportunity to understand the flow of material and information in CKD.

In a similar way, simple enumeration of EVs is not enough to establish a pathophysiological mechanism. Instead of it, time, place, and means of interactions (ligand binding, membrane fusion, or uptake by endocytosis), as well as identification of the specific molecular content(s) that mainly account for any given biological effect on target cells, are most critically connected to their desirable or harmful bioreactivity. However, this is also the hardest part in EV assessment. Incorporation of the guidelines suggested by the International Society for Extracellular Vesicles (Thery et al., 2018) would greatly benefit the emerging contemporary area of research on CKD-related EVs.

## Conclusion

Plasma EVs may have significant roles in CKD as disease biomarkers, risk factors for the development of complications, repair or protective factors diffusing harmful stress signals, and finally, therapeutic factors. They seem to be part of the problem and part of the solution. At times when advanced technologies for EVs assessment are still evolving, understanding of their biology, from biogenesis to ultimate effects on recipient cells, is of key importance to reveal their clinical relevance to CKD. Development of therapeutic interventions have already been oriented to both block their negative effects and take advantage of their beneficial properties. Regulation of EVs release or uptake by drugs, including statins, antioxidants, and actin depolymerizing agents, and removal from plasma by immunoadsorption have been reported (Karpman et al., 2017). Those interventions may allow prognosis, better diagnosis, and individualized treatments for CKD patients.

## Author Contributions

HG and MA conceived the concept, wrote the manuscript, and designed the figures. EPav, EPap, IP, and AK provided input and direction in structuring of the manuscript.

## Conflict of Interest

The authors declare that the research was conducted in the absence of any commercial or financial relationships that could be construed as a potential conflict of interest.
