# Supplementary material for: The Multi-Faced Extracellular Vesicles in the Plasma of Chronic Kidney Disease Patients
Source: Front Cell Dev Biol. 2020 Apr 15;8:227. doi: 10.3389/fcell.2020.00227 (PMC7174738; doi:10.3389/fcell.2020.00227)
Supplement: Supplementary file 1 [file Data_Sheet_1.PDF]

Research Articles (N=37)

1. Almquist T, Mobarrez F, Jacobson SH, Wallen H, Hjemdahl P. Effects of lipid-lowering treatment on circulating microparticles in patients with diabetes mellitus and chronic kidney disease. *Nephrol Dial Transplant* (2016) 31(6):944-52. doi: 10.1093/ndt/gfv337. PubMed PMID: 26394646.

2. Amabile N, Guerin AP, Leroyer A, Mallat Z, Nguyen C, Boddaert J, et al. Circulating endothelial microparticles are associated with vascular dysfunction in patients with end-stage renal failure. *J Am Soc Nephrol* (2005) 16(11):3381-8. Epub 2005/09/30. doi: ASN.2005050535 [pii] 10.1681/ASN.2005050535. PubMed PMID: 16192427.

3. Amabile N, Guerin AP, Tedgui A, Boulanger CM, London GM. Predictive value of circulating endothelial microparticles for cardiovascular mortality in end-stage renal failure: a pilot study. *Nephrol Dial Transplant* (2012) 27(5):1873-80. Epub 2011/11/01. doi: 10.1093/ndt/gfr573 gfr573 [pii]. PubMed PMID: 22036944.

4. Ando M, Iwata A, Ozeki Y, Tsuchiya K, Akiba T, Nihei H. Circulating platelet-derived microparticles with procoagulant activity may be a potential cause of thrombosis in uremic patients. *Kidney Int* (2002) 62(5):1757-63. Epub 2002/10/10. doi: kid627 [pii] 10.1046/j.1523-1755.2002.00627.x. PubMed PMID: 12371977.

5. Boulanger CM, Amabile N, Guerin AP, Pannier B, Leroyer AS, Mallat CN, et al. In vivo shear stress determines circulating levels of endothelial microparticles in end-stage renal disease. *Hypertension* (2007) 49(4):902-8. Epub 2007/02/21. doi: 01.HYP.0000259667.22309.df [pii] 10.1161/01.HYP.0000259667.22309.df. PubMed PMID: 17309952.

6. Burton JO, Hamali HA, Singh R, Abbasian N, Parsons R, Patel AK, et al. Elevated levels of procoagulant plasma microvesicles in dialysis patients. *PLoS One* (2013) 8(8):e72663. Epub 2013/08/13. doi: 10.1371/journal.pone.0072663 PONE-D-13-19511 [pii]. PubMed PMID: 23936542; PubMed Central PMCID: PMC3732282.

7. Carmona A, Aguera ML, Luna-Ruiz C, Buendia P, Calleros L, Garcia-Jerez A, et al. Markers of endothelial damage in patients with chronic kidney disease on hemodialysis. *Am J Physiol Renal Physiol* (2017) 312(4):F673-F81. doi: 10.1152/ajprenal.00013.2016. PubMed PMID: 28077371.

8. Cavallari C, Dellepiane S, Fonsato V, Medica D, Marengo M, Migliori M, et al. Online Hemodiafiltration Inhibits Inflammation-Related Endothelial Dysfunction and Vascular Calcification of Uremic Patients Modulating miR-223 Expression in Plasma Extracellular Vesicles. *J Immunol* (2019) 202(8):2372-83. doi: 10.4049/jimmunol.1800747. PubMed PMID: 30833349.

9. Daniel L, Fakhouri F, Joly D, Mouthon L, Nusbaum P, Grunfeld JP, et al. Increase of circulating neutrophil and platelet microparticles during acute vasculitis and hemodialysis. *Kidney Int* (2006) 69(8):1416-23. Epub 2006/03/15. doi: S0085-2538(15)51680-3 [pii] 10.1038/sj.ki.5000306. PubMed PMID: 16531979.

10. de Laval P, Mobarrez F, Almquist T, Vassil L, Fellstrom B, Soveri I. Acute effects of haemodialysis on circulating microparticles. *Clin Kidney J* (2019) 12(3):456-62. doi:

10.1093/ckj/sfy109. PubMed PMID: 31198549; PubMed Central PMCID: PMCPMC6543976.

11. Dursun I, Poyrazoglu HM, Gunduz Z, Ulger H, Yykylmaz A, Dusunsel R, et al. The relationship between circulating endothelial microparticles and arterial stiffness and atherosclerosis in children with chronic kidney disease. *Nephrol Dial Transplant* (2009) 24(8):2511-8. doi: 10.1093/ndt/gfp066. PubMed PMID: 19244228.

12. Farag YM, Keithy-Reddy SR, Mittal BV, Bansal V, Fareed J, Singh AK. Modulation of platelet activation in chronic kidney disease patients on erythropoiesis-stimulating agents. *Clin Appl Thromb Hemost* (2012) 18(5):453-61. doi: 10.1177/1076029611431954. PubMed PMID: 22496088. 10.1016/j.diabres.2004.10.010. PubMed PMID: 15936460.

13. Faure V, Dou L, Sabatier F, Cerini C, Sampol J, Berland Y, et al. Elevation of circulating endothelial microparticles in patients with chronic renal failure. *J Thromb Haemost* (2006) 4(3):566-73. Epub 2006/01/13. doi: JTH1780 [pii] 10.1111/j.1538-7836.2005.01780.x. PubMed PMID: 16405517.

14. Gao C, Ji S, Dong W, Qi Y, Song W, Cui D, et al. Indolic uremic solutes enhance procoagulant activity of red blood cells through phosphatidylserine exposure and microparticle release. *Toxins (Basel)* (2015) 7(11):4390-403. Epub 2015/11/01. doi: 10.3390/toxins7114390 toxins7114390 [pii]. PubMed PMID: 26516916; PubMed Central PMCID: PMC4663509.

15. Gao C, Xie R, Yu C, Ma R, Dong W, Meng H, et al. Thrombotic Role of Blood and Endothelial Cells in Uremia through Phosphatidylserine Exposure and Microparticle Release. *PLoS One* (2015) 10(11):e0142835. Epub 2015/11/19. doi: 10.1371/journal.pone.0142835 PONE-D-15-21475 [pii]. PubMed PMID: 26580207; PubMed Central PMCID: PMC4646287.

16. Georgatzakou HT, Tzounakas VL, Kriebardis AG, Velentzas AD, Papageorgiou EG, Voulgaridou AI, et al. Pathophysiological aspects of red blood cells in end-stage renal disease patients resistant to recombinant human erythropoietin therapy. *Eur J Haematol* (2017) 98(6):590-600. Epub 2017/03/16. doi: 10.1111/ejh.12875. PubMed PMID: 28295628.

17. Green D, Skeoch S, Alexander MY, Kalra PA, Parker B. The Association of Baseline and Longitudinal Change in Endothelial Microparticle Count with Mortality in Chronic Kidney Disease. *Nephron* (2017) 135(4):252-60. Epub 2017/01/25. doi: 10.1159/000452344 000452344 [pii]. PubMed PMID: 28118643.

18. Jalal D, Renner B, Laskowski J, Stites E, Cooper J, Valente K, et al. Endothelial Microparticles and Systemic Complement Activation in Patients With Chronic Kidney Disease. *J Am Heart Assoc* (2018) 7(14). doi: 10.1161/JAHA.117.007818. PubMed PMID: 30006493; PubMed Central PMCID: PMCPMC6064828.

19. Lau YC, Xiong Q, Blann AD, Lip GY. Relationship between renal function and circulating microparticles, soluble P-selectin and E-selectin levels in atrial fibrillation. *J Thromb Thrombolysis* (2017) 43(1):18-23. Epub 2016/09/28. doi: 10.1007/s11239-

016-1427-3 10.1007/s11239-016-1427-3 [pii]. PubMed PMID: 27671694; PubMed Central PMCID: PMC5233739.

20. Li S, Ren J, Xu N, Zhang J, Geng Q, Cao C, et al. MicroRNA-19b functions as potential anti-thrombotic protector in patients with unstable angina by targeting tissue factor. *J Mol Cell Cardiol* (2014) 75:49-57. doi: 10.1016/j.yjmcc.2014.06.017. PubMed PMID: 24998411.

21. Lu GY, Xu RJ, Zhang SH, Qiao Q, Shen L, Li M, et al. Alteration of circulatory platelet microparticles and endothelial microparticles in patients with chronic kidney disease. *Int J Clin Exp Med* (2015) 8(9):16704-8. Epub 2015/12/03. PubMed PMID: 26629207; PubMed Central PMCID: PMC4659095.

22. Lundwall K, Mortberg J, Mobarrez F, Jacobson SH, Jorneskog G, Spaak J. Changes in microparticle profiles by vitamin D receptor activation in chronic kidney disease - a randomized trial. *BMC Nephrol* (2019) 20(1):290. Epub 2019/08/03. doi: 10.1186/s12882-019-1445-4 10.1186/s12882-019-1445-4 [pii]. PubMed PMID: 31370809; PubMed Central PMCID: PMC6670162.

23. Martin N, Smith AC, Dungey MR, Young HML, Burton JO, Bishop NC. Exercise during hemodialysis does not affect the phenotype or prothrombotic nature of microparticles but alters their proinflammatory function. *Physiol Rep* (2018) 6(19):e13825. Epub 2018/10/09. doi: 10.14814/phy2.13825. PubMed PMID: 30294974; PubMed Central PMCID: PMC6174123.

24. Merino A, Portoles J, Selgas R, Ojeda R, Buendia P, Ocana J, et al. Effect of different dialysis modalities on microinflammatory status and endothelial damage. *Clin J Am Soc Nephrol* (2010) 5(2):227-34. doi: 10.2215/CJN.03260509. PubMed PMID: 20056757; PubMed Central PMCID: PMCPMC2827586.

25. Ogata N, Imaizumi M, Nomura S, Shozu A, Arichi M, Matsuoka M, et al. Increased levels of platelet-derived microparticles in patients with diabetic retinopathy. *Diabetes Res Clin Pract* (2005) 68(3):193-201. Epub 2005/06/07. doi: S0168-8227(04)00311-0

26. Ogata N, Nomura S, Shouzu A, Imaizumi M, Arichi M, Matsumura M. Elevation of monocyte-derived microparticles in patients with diabetic retinopathy. *Diabetes Res Clin Pract* (2006) 73(3):241-8. Epub 2006/04/06. doi: S0168-8227(06)00056-8 [pii] 10.1016/j.diabres.2006.01.014. PubMed PMID: 16584800.

27. Pan Y, Liang H, Liu H, Li D, Chen X, Li L, et al. Platelet-secreted microRNA-223 promotes endothelial cell apoptosis induced by advanced glycation end products via targeting the insulin-like growth factor 1 receptor. *J Immunol* (2014) 192(1):437-46. doi: 10.4049/jimmunol.1301790. PubMed PMID: 24307738.

28. Qamri Z, Pelletier R, Foster J, Kumar S, Momani H, Ware K, et al. Early posttransplant changes in circulating endothelial microparticles in patients with kidney transplantation. *Transpl Immunol* (2014) 31(2):60-4. Epub 2014/07/11. doi: 10.1016/j.trim.2014.06.006 S0966-3274(14)00049-5 [pii]. PubMed PMID: 25008980; PubMed Central PMCID: PMC4141008.

29. Rodrigues KF, Pietrani NT, Fernandes AP, Bosco AA, de Sousa MCR, de Fatima Oliveira Silva I, et al. Circulating microparticles levels are increased in patients with

diabetic kidney disease: A case-control research. *Clin Chim Acta* (2018) 479:48-55. Epub 2018/01/07. doi: S0009-8981(17)30548-X [pii] 10.1016/j.cca.2017.12.048. PubMed PMID: 29305843.

30. Ruzicka M, Xiao F, Abujrad H, Al-Rewashdy Y, Tang VA, Langlois MA, et al. Effect of hemodialysis on extracellular vesicles and circulating submicron particles. *BMC Nephrol* (2019) 20(1):294. doi: 10.1186/s12882-019-1459-y. PubMed PMID: 31375072; PubMed Central PMCID: PMCPMC6679543.

31. Ryu JH, Lim SY, Ryu DR, Kang DH, Choi KB, Kim SJ. Association between vascular access failure and microparticles in hemodialysis patients. *Kidney Res Clin Pract* (2012) 31(1):38-47. doi: 10.1016/j.krcp.2011.12.002. PubMed PMID: 26889407; PubMed Central PMCID: PMCPMC4715093.

32. Soriano S, Carmona A, Trivino F, Rodriguez M, Alvarez-Benito M, Martin-Malo A, et al. Endothelial damage and vascular calcification in patients with chronic kidney disease. *Am J Physiol Renal Physiol* (2014) 307(11):F1302-11. Epub 2014/10/24. doi: 10.1152/ajprenal.00114.2014 ajprenal.00114.2014 [pii]. PubMed PMID: 25339701.

33. Trappenburg MC, van Schilfgaarde M, Frerichs FC, Spronk HM, ten Cate H, de Fijter CW, et al. Chronic renal failure is accompanied by endothelial activation and a large increase in microparticle numbers with reduced procoagulant capacity. *Nephrol Dial Transplant* (2012) 27(4):1446-53. Epub 2011/08/30. doi: 10.1093/ndt/gfr474 gfr474 [pii]. PubMed PMID: 21873622.

34. Viegas C, Araujo N, Marreiros C, Simes D. The interplay between mineral metabolism, vascular calcification and inflammation in Chronic Kidney Disease (CKD): challenging old concepts with new facts. *Aging (Albany NY)* (2019) 11(12):4274-99. Epub 2019/06/27. doi: 10.18632/aging.102046 102046 [pii]. PubMed PMID: 31241466; PubMed Central PMCID: PMC6628989.

35. Xie JX, Fan X, Drummond CA, Majumder R, Xie Y, Chen T, et al. MicroRNA profiling in kidney disease: Plasma versus plasma-derived exosomes. *Gene* (2017) 627:1-8. doi: 10.1016/j.gene.2017.06.003. PubMed PMID: 28587849; PubMed Central PMCID: PMCPMC5534180.

36. Yu M, Xie R, Zhang Y, Liang H, Hou L, Yu C, et al. Phosphatidylserine on microparticles and associated cells contributes to the hypercoagulable state in diabetic kidney disease. *Nephrol Dial Transplant* (2018) 33(12):2115-27. doi: 10.1093/ndt/gfy027. PubMed PMID: 29529237.

37. Zhang Y, Ma KL, Gong YX, Wang GH, Hu ZB, Liu L, et al. Platelet Microparticles Mediate Glomerular Endothelial Injury in Early Diabetic Nephropathy. *J Am Soc Nephrol* (2018) 29(11):2671-95. doi: 10.1681/ASN.2018040368. PubMed PMID: 30341150; PubMed Central PMCID: PMCPMC6218868.

Review Articles (N=26)

1. Abbasian N, Herbert KE, Pawluczyk I, Burton JO, Bevington A. Vesicles bearing gifts: the functional importance of micro-RNA transfer in extracellular vesicles in chronic kidney disease. *Am J Physiol Renal Physiol* (2018) 315(5):F1430-F43. Epub 2018/08/16. doi: 10.1152/ajprenal.00318.2018. PubMed PMID: 30110570.

2. Borges FT, Reis LA, Schor N. Extracellular vesicles: structure, function, and potential clinical uses in renal diseases. *Braz J Med Biol Res* (2013) 46(10):824-30. Epub 2013/10/22. doi: 10.1590/1414-431X20132964 S0100-879X2013005032964 [pii]. PubMed PMID: 24141609; PubMed Central PMCID: PMC3854311.

3. Carney EF. Chronic kidney disease: Procoagulant microparticles provide a novel pathogenic link between hyperphosphataemia and cardiovascular risk. *Nat Rev Nephrol* (2015) 11(5):256. Epub 2015/03/25. doi: 10.1038/nrneph.2015.34 nrneph.2015.34 [pii]. PubMed PMID: 25802076.

4. Chironi GN, Boulanger CM, Simon A, Dignat-George F, Freyssinet JM, Tedgui A. Endothelial microparticles in diseases. *Cell Tissue Res* (2009) 335(1):143-51. Epub 2008/11/08. doi: 10.1007/s00441-008-0710-9. PubMed PMID: 18989704.

5. Chung AC. microRNAs in Diabetic Kidney Disease. Adv Exp Med Biol (2015) 888:253-69. doi: 10.1007/978-3-319-22671-2_13. PubMed PMID: 26663187.

6. Clementi A, Virzi GM, Battaglia GG, Ronco C. Neurohormonal, Endocrine, and Immune Dysregulation and Inflammation in Cardiorenal Syndrome. *Cardiorenal Med* (2019) 9(5):265-73. Epub 2019/07/03. doi: 10.1159/000500715 000500715 [pii]. PubMed PMID: 31266023.

7. Console L, Scalise M, Indiveri C. Exosomes in inflammation and role as biomarkers. *Clin Chim Acta* (2019) 488:165-71. Epub 2018/11/13. doi: S0009-8981(18)30581-3 [pii] 10.1016/j.cca.2018.11.009. PubMed PMID: 30419221.

8. Daniel L, Dou L, Berland Y, Lesavre P, Mecarelli-Halbwachs L, Dignat-George F. Circulating microparticles in renal diseases. *Nephrol Dial Transplant* (2008) 23(7):2129-32. Epub 2008/02/19. doi: 10.1093/ndt/gfn029 gfn029 [pii]. PubMed PMID: 18281318.

9. Dursun I, Yel S, Unsur E. Dynamics of circulating microparticles in chronic kidney disease and transplantation: Is it really reliable marker? *World J Transplant* (2015) 5(4):267-75. Epub 2016/01/02. doi: 10.5500/wjt.v5.i4.267. PubMed PMID: 26722654; PubMed Central PMCID: PMC4689937.

10. Erdbrugger U, Le TH. Extracellular Vesicles in Renal Diseases: More than Novel Biomarkers? *J Am Soc Nephrol* (2016) 27(1):12-26. Epub 2015/08/08. doi: 10.1681/ASN.2015010074 ASN.2015010074 [pii]. PubMed PMID: 26251351; PubMed Central PMCID: PMC4696584.

11. Favretto G, Cunha RSD, Dalboni MA, Oliveira RB, Barreto FC, Massy ZA, et al. Endothelial Microparticles in Uremia: Biomarkers and Potential Therapeutic Targets. *Toxins (Basel)* (2019) 11(5). Epub 2019/05/16. doi: E267 [pii] 10.3390/toxins11050267 toxins11050267 [pii]. PubMed PMID: 31086003; PubMed Central PMCID: PMC6563302.

12. Goettsch C, Hutcheson JD, Aikawa E. MicroRNA in cardiovascular calcification: focus on targets and extracellular vesicle delivery mechanisms. *Circ Res* (2013) 112(7):1073-84. Epub 2013/03/30. doi: 10.1161/CIRCRESAHA.113.300937 112/7/1073 [pii]. PubMed PMID: 23538277; PubMed Central PMCID: PMC3668680.

13. Helmke A, von Vietinghoff S. Extracellular vesicles as mediators of vascular inflammation in kidney disease. *World J Nephrol* (2016) 5(2):125-38. Epub 2016/03/17. doi: 10.5527/wjn.v5.i2.125. PubMed PMID: 26981436; PubMed Central PMCID: PMC4777783.

14. Jing H, Tang S, Lin S, Liao M, Chen H, Zhou J. The role of extracellular vesicles in renal fibrosis. *Cell Death Dis* (2019) 10(5):367. Epub 2019/05/10. doi: 10.1038/s41419-019-1605-2 10.1038/s41419-019-1605-2 [pii]. PubMed PMID: 31068572; PubMed Central PMCID: PMC6506498.

15. Jourde-Chiche N, Dou L, Cerini C, Dignat-George F, Brunet P. Vascular incompetence in dialysis patients--protein-bound uremic toxins and endothelial dysfunction. *Semin Dial* (2011) 24(3):327-37. Epub 2011/06/21. doi: 10.1111/j.1525-139X.2011.00925.x. PubMed PMID: 21682773.

16. Karpman D, Stahl AL, Arvidsson I. Extracellular vesicles in renal disease. *Nat Rev Nephrol* (2017) 13(9):545-62. Epub 2017/07/25. doi: 10.1038/nrneph.2017.98 nrneph.2017.98 [pii]. PubMed PMID: 28736435.

17. Lu CC, Ma KL, Ruan XZ, Liu BC. The Emerging Roles of Microparticles in Diabetic Nephropathy. *Int J Biol Sci* (2017) 13(9):1118-25. Epub 2017/11/07. doi: 10.7150/ijbs.21140 ijbsv13p1118 [pii]. PubMed PMID: 29104503; PubMed Central PMCID: PMC5666327.

18. Lutz J, Menke J, Sollinger D, Schinzel H, Thurmel K. Haemostasis in chronic kidney disease. *Nephrol Dial Transplant* (2014) 29(1):29-40. Epub 2013/10/18. doi: 10.1093/ndt/gft209 gft209 [pii]. PubMed PMID: 24132242.

19. Lv LL, Feng Y, Tang TT, Liu BC. New insight into the role of extracellular vesicles in kidney disease. *J Cell Mol Med* (2019) 23(2):731-9. Epub 2018/12/27. doi: 10.1111/jcmm.14101. PubMed PMID: 30585399; PubMed Central PMCID: PMC6349185.

20. Mohandas R, Segal MS. Endothelial progenitor cells and endothelial vesicles - what is the significance for patients with chronic kidney disease? *Blood Purif* (2010) 29(2):158-62. Epub 2010/01/23. doi: 10.1159/000245643 000245643 [pii]. PubMed PMID: 20093822; PubMed Central PMCID: PMC2914407.

21. Quinn JF, Patel T, Wong D, Das S, Freedman JE, Laurent LC, et al. Extracellular RNAs: development as biomarkers of human disease. *J Extracell Vesicles* (2015) 4:27495. Epub 2015/09/01. doi: 10.3402/jev.v4.27495 27495 [pii]. PubMed PMID: 26320940; PubMed Central PMCID: PMC4553262.

22. Rabelink TJ, de Boer HC, van Zonneveld AJ. Endothelial activation and circulating markers of endothelial activation in kidney disease. *Nat Rev Nephrol* (2010) 6(7):404-14. Epub 2010/05/26. doi: 10.1038/nrneph.2010.65 nrneph.2010.65 [pii]. PubMed PMID: 20498676.

23. Stahl AL, Johansson K, Mossberg M, Kahn R, Karpman D. Exosomes and microvesicles in normal physiology, pathophysiology, and renal diseases. *Pediatr Nephrol* (2019) 34(1):11-30. doi: 10.1007/s00467-017-3816-z. PubMed PMID: 29181712; PubMed Central PMCID: PMCPMC6244861.

24. Viegas C, Araujo N, Marreiros C, Simes D. The interplay between mineral metabolism, vascular calcification and inflammation in Chronic Kidney Disease (CKD): challenging old concepts with new facts. *Aging (Albany NY)* (2019) 11(12):4274-99. Epub 2019/06/27. doi: 10.18632/aging.102046 102046 [pii]. PubMed PMID: 31241466; PubMed Central PMCID: PMC6628989.

25. Zhang W, Chen S, Liu ML. Pathogenic roles of microvesicles in diabetic retinopathy. *Acta Pharmacol Sin* (2018) 39(1):1-11. Epub 2017/07/18. doi: 10.1038/aps.2017.77 aps201777 [pii]. PubMed PMID: 28713160; PubMed Central PMCID: PMC5758669.

26. Zhang W, Zhou X, Zhang H, Yao Q, Liu Y, Dong Z. Extracellular vesicles in diagnosis and therapy of kidney diseases. *Am J Physiol Renal Physiol* (2016) 311(5):F844-F51. Epub 2016/11/03. doi: 10.1152/ajprenal.00429.2016 ajprenal.00429.2016 [pii]. PubMed PMID: 27582107; PubMed Central PMCID: PMC5130456.
